# Supplementary material for: Effects of Fatty Acid Metabolites on Adipocytes Britening: Role of Thromboxane A2
Source: Cells. 2023 Jan 30;12(3):446. doi: 10.3390/cells12030446 (PMC9913700; doi:10.3390/cells12030446)
Supplement: Supplementary file 1 [file cells-12-00446-s001.zip › cells-2132867-supplementary.pdf]

Supporting Information

Materials and Methods

Mouse Adipose Tissue SVF Preparation and Culture

Twelve-week-old C57Bl/6J mice were euthanized by cervical dislocation to isolate stromal vascular fractions (SVF) as described previously [38,60–62]. Briefly, inguinal subcutaneous white adipose tissue and interscapular brown adipose tissue depots were sampled, washed in PBS, and minced. Adipose tissue samples were digested for 45 min at 37°C in DMEM containing 2 mg/ml collagenase A (Roche Diagnostics, Meylan, France) and 20 mg/ml bovine serum albumin (Sigma-Aldrich Chimie, Saint-Quentin Fallavier, France). Cell lysates were successively filtered through 250, 100 and 27 µm nylon sheets, and centrifuged for 5 min at 500 g at room temperature. The pellet containing cells of the stromal vascular fraction (SVF) was subjected to a red blood cell lysis procedure. SVF cells were plated and maintained in DMEM containing 10% FCS until confluence, designed as day 0. Differentiation was induced in the same medium supplemented with 1 µM dexamethasone, 0.5 mM isobutylmethylxanthine, 2 nM triiodothyronine and 170 nM insulin for two days, between days 0 and 2. Cells were then maintained for 8-10 days in DMEM containing 10% FCS in the presence of 10 nM insulin, 2 nM triiodothyronine and 1 µM Rosiglitazone for brown adipogenesis. Media were changed every other day.

References

60. Rodbell, M. Metabolism of Isolated Fat Cells. I. Effects of Hormones on Glucose Metabolism and Lipolysis. *J. Biol. Chem.* **1964**, 239, 375-380

61. Murholm, M., Dixen, K., Qvortrup, K., Hansen, L. H., Amri, E. Z., Madsen, L., Barbatelli, G., Quistorff, B., and Hansen, J. B. Dynamic regulation of genes involved in mitochondrial DNA replication and transcription during mouse brown fat cell differentiation and recruitment. *PLoS One* **2009**, 4, e8458,10.1371/journal.pone.0008458

38. Ghandour, R. A., Giroud, M., Vegiopoulos, A., Herzig, S., Ailhaud, G., Amri, E. Z., and Pisani, D. F. IP-receptor and PPARs trigger the conversion of human white to brite adipocyte induced by carbaprostacyclin. *Biochim. Biophys. Acta* **2016**, 1861, 285-293,10.1016/j.bbalip.2016.01.007

62. Colson, C., Batrow, P. L., Gautier, N., Rochet, N., Ailhaud, G., Peiretti, F., and Amri, E. Z. The Rosmarinus Bioactive Compound Carnosic Acid Is a Novel PPAR Antagonist That Inhibits the Browning of White Adipocytes. *Cells* **2020**, 9,10.3390/cells9112433

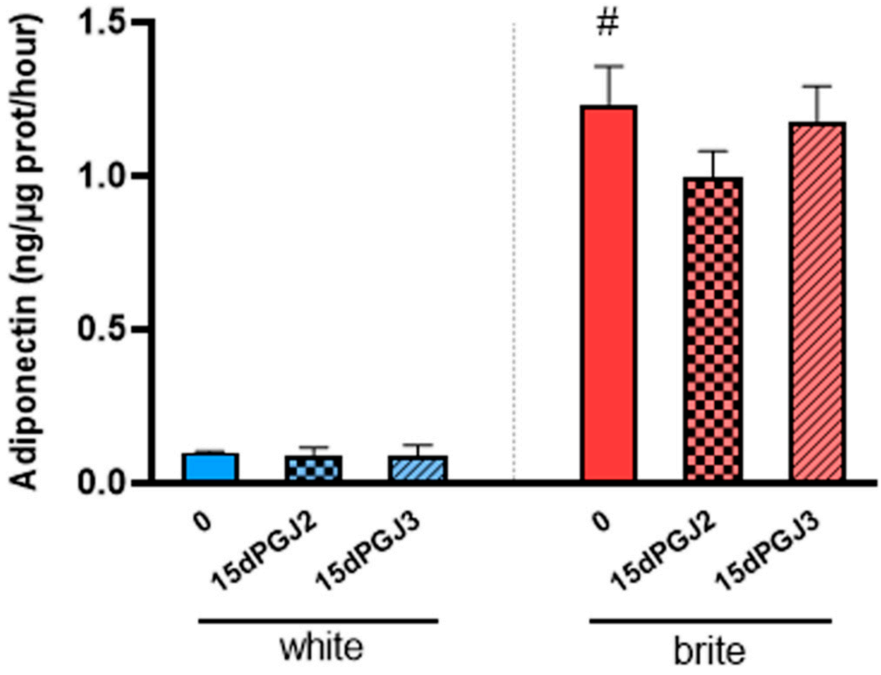

Figure S1. Adiponectin secretion

White hMADS adipocytes (blue columns) were induced to convert (from day 14 to day 18) into brite adipocytes (red columns) in the presence of 0.5 µM 15-deoxy-delta 12,14-PGJ2 or 15-deoxy-delta 12,14-PGJ3 in the absence or the presence of 100 nM rosiglitazone. Adiponectin levels were measured in the media six hours after the last medium change. Histograms display mean ± SEM of three independent experiments; Paired student, $p<0.05$ considered as significant: #, white vs brite adipocyte.

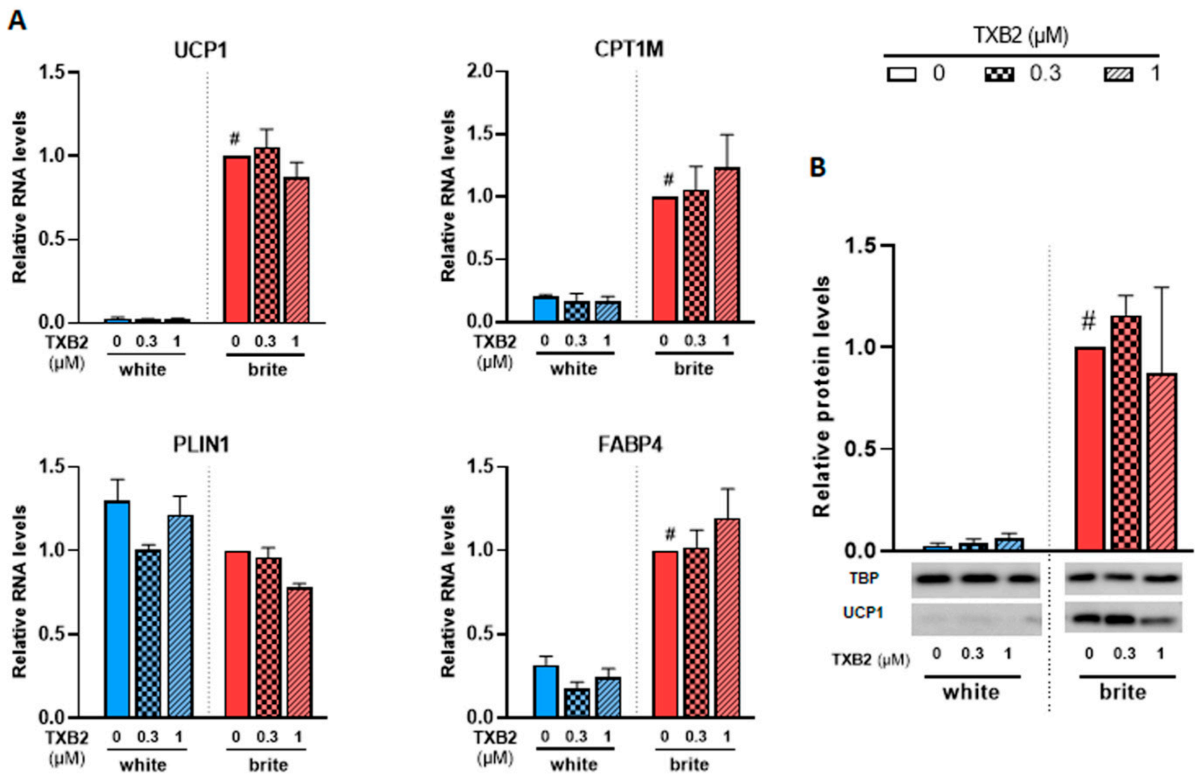

Figure S2. TXB2 did not affect UCP1 expression. White hMADS adipocytes (blue columns) were induced to convert (from day 14 to day 18) into brite adipocytes (red columns) in the presence of various amounts of TXB2 in the absence (white) or the presence of 100 nM rosiglitazone (brite). UCP1, CPT1M, PLIN1 and FABP4 mRNA levels were measured (A). 40 µg total protein extracts were analyzed by Western blot (B). Histograms display mean ± SEM of three to four (A) or two (B) independent experiments; Statistics by Two-way ANOVA with Tukey's multiple comparisons test, $p<0.05$ considered as significant: #, white vs brite adipocyte.

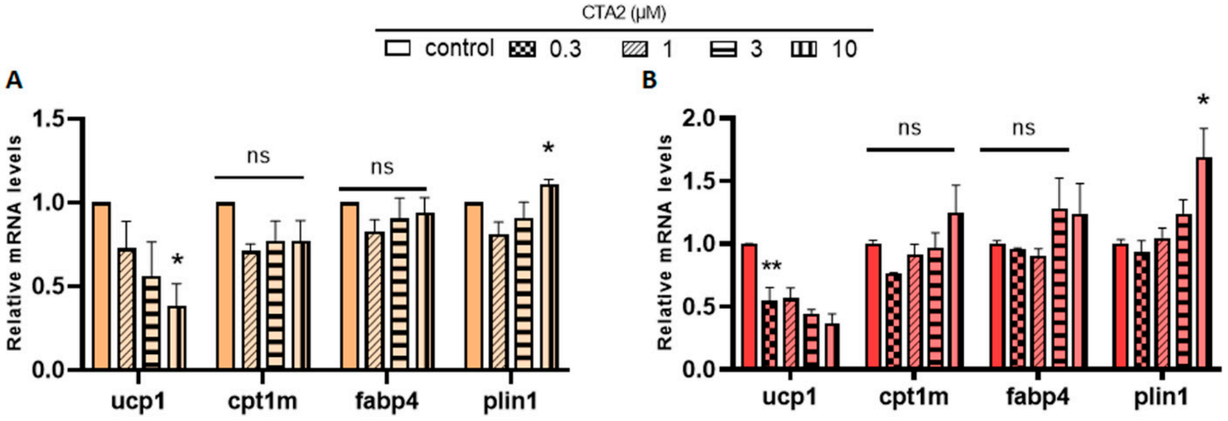

Figure S3. Effects of CTA2 on the browning process of mice primary adipocytes. SVF cells derived from mice subcutaneous (A) and brown adipose (B) tissues were induced to differentiate into brown (rosiglitazone-treatment) adipocytes in the absence or presence of various amounts of CT2A for the last 4 days and mRNA levels of thermogenic and adipogenic markers were analyzed. Histograms display mean ± SEM of two to four independent experiments. Statistics by One-way ANOVA with Dunett's multiple comparisons test, $p<0.05$ considered as significant: *, **, treated vs untreated.

Table S1. Sequence of primers used for gene expression analysis.

name	Reverse primer	Forward primer
human		
PPARγ2	ATCAGTGAAGGAATCGCTTTCTG	CAAACCCCTATTCCATGCTGTT
UCP1	CCAGGATCCAAGTCGCAAGA	GTGTGCCCAACTGTGCAATG
FABP4	CAACGTCCCTTGGCTTATGCT	TGTGCAGAAATGGGATGGAAA
CPT1-M	GAGCAGCACCCCAATCAC	AACTCCATAGCCATCATCTGCT
PLN1	GATGGGAACGCTGATGCTGTT	ACCCCCCTGAAAAGATTGCTT
36B4	TGCATCAGTACCCCATTCTATCAT	AGGCAGATGGATCAGCCAAGA
PTGIR	TAGGTGAGGTTCCTGCACGAA	AGCCTGGGCAAGACTGGAG
TXA2R	CACTGTCTGGGCGATGAAGA	CGAGGTGGAGATGATGGCTC
Mice		
ucp1	CCTGGCCTTCACCTTGGAT	CACCTTCCCGCTGGACACT
36b4	TCCAGGCTTTGGGCATCA	CTTTATCAGCTGCACATCACTCAGA
plin	CTTCTGGAAGCACTCACAGG	AGCGTGGAGAGTAAGGATGTC
cpt1m	GGCTCCAGGGTTCAGAAAGT	AAACAACGCAACGTGGAGA
fabp4	CTTGTGGAAGTCACGCCTTT	AAGAGAAAACGAGATGGTGACAA
